# Simulation-Based Estimates of Effectiveness and Cost-Effectiveness of Smoking Cessation in Patients with Chronic Obstructive Pulmonary Disease

**DOI:** 10.1371/journal.pone.0024870

**Published:** 2011-09-14

**Authors:** Kokuvi Atsou, Christos Chouaid, Gilles Hejblum

**Affiliations:** 1 INSERM, U707, Paris, France; 2 UPMC Univ Paris 06, UMR S 707, Paris, France; 3 AP-HP, Hôpital Saint Antoine, Service de Pneumologie, Paris, France; 4 AP-HP, Hôpital Saint Antoine, Unité de Santé Publique, Paris, France; Genentech Inc., United States of America

## Abstract

**Background:**

The medico-economic impact of smoking cessation considering a smoking patient with chronic obstructive pulmonary disease (COPD) is poorly documented.

**Objective:**

Here, considering a COPD smoking patient, the specific burden of continuous smoking was estimated, as well as the effectiveness and the cost-effectiveness of smoking cessation.

**Methods:**

A multi-state Markov model adopting society's perspective was developed. Simulated cohorts of English COPD patients who are active smokers (all severity stages combined or patients with the same initial severity stage) were compared to identical cohorts of patients who quit smoking at cohort initialization. Life expectancy, quality adjusted life-years (QALY), disease-related costs, and incremental cost-effectiveness ratio (ICER: £/QALY) were estimated, considering smoking cessation programs with various possible scenarios of success rates and costs. Sensitivity analyses included the variation of model key parameters.

**Principal Findings:**

At the horizon of a smoking COPD patient's remaining lifetime, smoking cessation at cohort intitialization, relapses being allowed as observed in practice, would result in gains (mean) of 1.27 life-years and 0.68 QALY, and induce savings of −1824 £/patient in the disease-related costs. The corresponding ICER was −2686 £/QALY. Smoking cessation resulted in 0.72, 0.69, 0.64 and 0.42 QALY respectively gained per mild, moderate, severe, and very severe COPD patient, but was nevertheless cost-effective for mild to severe COPD patients in most scenarios, even when hypothesizing expensive smoking cessation intervention programmes associated with low success rates. Considering a ten-year time horizon, the burden of continuous smoking in English COPD patients was estimated to cost a total of 1657 M£ while 452516 QALY would be simultaneously lost.

**Conclusions:**

The study results are a useful support for the setting of smoking cessation programmes specifically targeted to COPD patients.

## Introduction

Chronic obstructive pulmonary disease (COPD) is a major cause of disability and mortality worldwide. COPD morbidity and mortality are expected to rise substantially in coming decades, and COPD is predicted to become the fourth leading cause of death by 2030 [1]. In industrialised countries, smoking is the main risk factor for COPD [2]. Once COPD is established, smoking is also associated with more rapid disease progression: pulmonary function declines twice as fast in continuing COPD smokers than in sustained COPD quitters [3]. Society's view of smoking has changed profoundly in recent decades [4], leading to prevention campaigns and legal measures aiming at reducing tobacco consumption [5], and there are now both societal and personal reasons why COPD smokers should quit. However, many such patients consider they are too old to benefit from smoking cessation, or that the damage to their health is already inexorable [6]. Shahab et al estimated that COPD smokers represent 4.6% of the English population over 35 years of age [7].

The cost-effectiveness of smoking cessation programmes has been thoroughly studied (see for example [8]), but few studies have focused on the medico-economic impact of changes in the smoking status of COPD patients. Only two studies have estimated the cost-effectiveness of smoking cessation programmes for COPD patients at the population scale [9], [10]. Based on a model including the future estimated cases of COPD over the next 25 years in the Netherlands, these studies indicated that smoking cessation programmes would be cost-effective. In particular, a smoking cessation programme combining intensive counseling and pharmacotherapy with a 12.3% 12-month abstinence rate and applied to 50% of the cohort of Dutch COPD smokers would result in an incremental cost-effectiveness ratio (ICER) of 2400 € per quality adjusted life-year (QALY) gained, as compared to usual care [9]. Here, a patient-centered model adapted to English COPD smokers was developed for estimating the impact of smoking cessation according to disease severity. A cohort of such patients was simulated, and the patients were followed-up until death. In a first step, the aim was to estimate what a COPD smoker who stops smoking should expect in terms of health gains: surprisingly, the impact of smoking cessation on such a patient's life expectancy and QALYs has never been reported. In a second step, we included costs of the disease and performed a cost-effectiveness study exploring several scenarios with the impact of smoking cessation programmes having different costs and efficacies, and applied to patients at different moments of disease progression.

## Methods

The study adopted society's viewpoint and the time horizon extended from initial smoking cessation by an English COPD patient to his or her death (all-cause mortality in COPD patients). All costs are expressed in 2010 pounds Sterling (£) and health outcomes are expressed as life-years (LY) and QALY. As recommended by the National Institute for Health and Clinical Excellence (NICE) [11], all costs and QALY values were discounted at a rate of 3.5%. The cost-effectiveness criterion for a given scenario was the incremental cost-effectiveness ratio (ICER), i.e. the ratio of the difference in costs between the intervention and non intervention to the corresponding difference in QALY, as recommended by international guidelines [12].

### Intervention

In a first step, this study estimated the burden of continuous smoking: An hypothetical cohort of standard English COPD continuous smokers cohort was simulated and compared to a similar hypothetical cohort of patients who all stop smoking at cohort initialization and remain sustained quitters. To analyse the potential difference in the continuous smoking according to COPD severity, we also simulated cohorts in which all the patients had the same initial severity.

In a second step, the study explored in detail the impact of smoking cessation, whether following a specific intervention programme where various success rate and intervention cost were considered (see also further details below in Sensitivity Analysis) or not: the above-mentioned continuous smokers' cohort (further referred to as “no intervention”) was compared to a similar hypothetical cohort where a proportion of patients stop smoking at cohort initialization (further referred to as “intervention”), but a yearly turnover of smoking status was allowed in both cohorts. The comparison of these two cohorts is further referred to as the “reference case”, and allows estimating the impact of smoking cessation in a typical COPD smoker. To analyse the potential difference in the impact of smoking cessation according to COPD severity, we also simulated cohorts in which all the patients had the same initial severity.

### Monte-Carlo Simulation of a cohort of English COPD smokers

Multi-state Markov modeling is a useful tool for representing a given disease [13]. Evolution of the disease in a cohort of patients is reflected by the changes in the relative proportions of patients in the different states along time. The time horizon of the analysis is divided into equal increments of time (e.g. year) and at each cycle, the process of transitions from one state to another (e.g. mild to moderate, or mild to death) is repeated, resulting in a given proportion of patients in each state at a given time. Such transitions as well as events that might influence the transitions (e.g. death not only depends on the grading of the disease but also on age, transitions rates may depend on smoking status, …) depend on probabilities that might vary with time (e.g. age-specific mortality). There are two ways of studying a Markov model, the cohort simulation and the Monte Carlo simulation. In the cohort simulation, all individuals are considered as a whole, and one only directly calculates the expected proportions of patients in the different states along time. In the Monte Carlo simulation approach, each patient of the cohort is individually simulated, each event being randomly chosen thanks to a pseudo-random number generator. Therefore, Monte Carlo simulations have the advantage of providing not only expected values, but allowing the examination of the associated variability.

**Figure 1:**
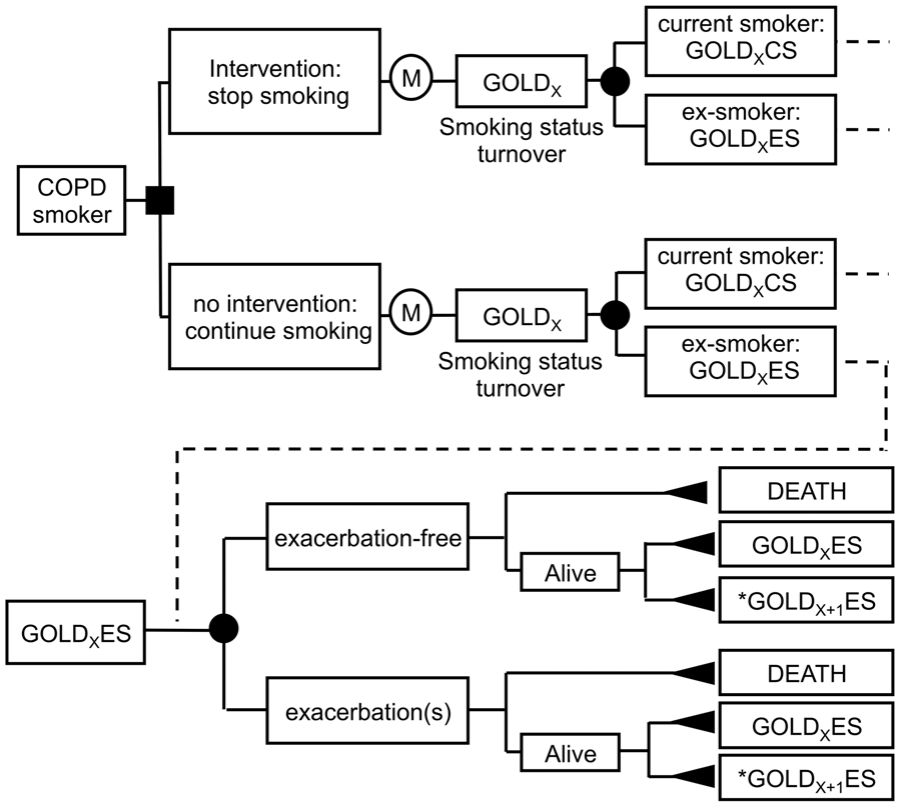
Flowchart describing the Markov multi-state model used. X value is 1, 2, 3, or 4, respectively corresponding to GOLD 1, GOLD 2, GOLD 3 and GOLD 4 stages. Each of these healthcare states is associated with a corresponding utility and cost. ^*^Value “X+1” does not exist for X = 4 (stage GOLD4). Nodes marked with an “M” represent Markov process chance nodes, while full square, full circle, and full triangle nodes correspond to decision, chance, and terminal nodes, respectively. Evolution of the cohort is made with one-year iteration step. Each patient is followed until death (all causes of death in COPD patients). At each iteration (Markov node), a given patient in a given X severity stage, is first subjected to a potential change in his/her smoking status, reflecting the background turnover observed in COPD patients (top). As indicated in Table 1, turnover probabilities were constant over age and COPD severity stages. Then (bottom), he might experience exacerbations (that only depend on patient's COPD current severity stage, as indicated in Table 1). In the end, the patient may 1) stay in the same severity stage, 2) pass to the next severity stage (X+1), 3) die. Transition probabilities from one stage to the next depend on age, severity stage, and smoking status (see parameter values in Table S2 in supporting information). Transition probabilities to death depend on the same parameters and in addition, on exacerbation status (see parameter values in in Table S3 in supporting information). As compared to current smokers, ex-smokers had a lower disease progression, and a lower probability of death.

We developed a multi-state Markov model based on the natural history of COPD (Figure 1). Using Monte Carlo simulation, each patient in the cohort was simulated until death (maximum age was set at 110 years). Each iteration was a one-year period during which the patient might either die (death was the final and absorbing state) or being subjected to a transition rate from his current severity stage to the next. Parameter values used in the model are summarized in Table 1 and are detailed below.

**Table 1:** Parameter values used in the Monte-Carlo simulations (reference case).

Parameter	COPD severity stage			
	GOLD1	GOLD2	GOLD3	GOLD4
COPD severity distribution (% of patients)	35.08	48.17	13.96	2.79
Annual smoking transition rates (% of patients)				
Quit smoking	4.7 whatever the severity stage			
Resume smoking	2.6 whatever the severity stage			
Exacerbation rates (% of patients) [0 exacerbation ; ≥1 exacerbation]	[75.00; 25.00]	[60.55; 39.45]	[55.90; 44.10]	[34.30; 65.70]
Age distribution per severity stage	Based on references 7 and 15; see supporting information, Table S1			
Annual transition rate to next severity stage	Based on references 10 and 17; see supporting information, Table S2			
Annual mortality rate per severity stage	Based on references 20 and 21; see supporting information, Table S3			
Health utility (QALY) [0 exacerbation ; ≥1 exacerbation]	[0.897; 0.895]	[0.755; 0.736]	[0.748; 0.726]	[0.549; 0.535]
Annual cost of COPD (£/patient)*	220	726	3758	9470

COPD, chronic obstructive pulmonary disease; QALY, quality adjusted life-years.

*The COPD annual cost included direct (drugs. hospitalization. outpatient care. equipment aids. oxygen therapy) and indirect cost (disability pensions. absence from work).

In a simulated cohort, a patient enters with a given COPD severity state, age, and smoking status. The severity states were those of the Global Initiative for Chronic Obstructive Lung Disease [14], namely GOLD1, GOLD 2, GOLD 3, GOLD 4. Shahab et al have reported the distribution of the English COPD patients in stage GOLD1, GOLD2, and GOLD3+GOLD4 according to smoking status [7]. As reported for Sweden [15], we postulated that respectively 5/6 and 1/6 patients were at stages GOLD3 and GOLD4. Shahab reported prevalence data for 10-year age classes; we considered that the age distributions in stages GOLD3 and GOLD4 were identical, and that the prevalence of COPD was uniform within each 10-year age class.

The initial distributions of age according to severity stages were set by adjusting Shahab's prevalence data to age distribution in England, as issued from the Office For National Statistics [16]. Thus, an initial cohort of individuals aged from 40 to 89 years was created (see supporting information, Table S1), likely to reflect the current distribution of English COPD smokers.

All simulations were performed with TreeAge Pro software (© 2007, TreeAge Software, Inc. Williamstown, MA, USA). Each simulated cohort was composed of 10^6^ patients.

### Disease progression parameter values: transitions from one severity stage to the next and exacerbation rates

The transition rates from a given severity stage to the next during a one-year iteration depended on the current severity stage, patient age, and smoking status. As detailed in the supporting information (Table S2), the corresponding transition probability table was built by combining transition probabilities from the Framingham cohort, as reported by Nielsen et al. [17], with transition probabilities according to smoking status, as reported by Hoogendoorn et al. [10]. The probability of experiencing one or more exacerbations was chosen according to Soler-Cataluna et al. [18] and depended on the severity stage.

### Disease progression parameter values: probability of death

The specific mortality table used in the present study simulations is detailed in Table S3 of the supporting information. The table was built using three information sources: all-cause mortality data (codes A00-Y89) for the UK general population in 2007 [19], data from Mannino et al [20] for taking into account excess mortality associated with COPD, and data from Ekberg-Aronsson et al. [21] for taking into account the smoking status of COPD patients.

### Disease progression parameter values: smoking status

A first particular scenario compared a simulated cohort where all COPD patients remain continuous smokers during their remaining lifetime to an identical simulated cohort of COPD patients who quit smoking at simulation initiation and remain ex-smokers during their remaining lifetime. However, although such a particular scenario allows estimating the raw impact of continuous smoking in COPD patients, all other simulations were conducted assuming quit and relapse events to occur during a patient remaining lifetime, as observed in practice: during a one-year iteration, the probabilities of smoking cessation by smokers and of relapse by ex-smokers were respectively set to 0.047 and 0.026, according to Hoogendoorn et al. [10]. These probabilities were constant over time, whatever the patient's age and severity stage. Importantly, these quit and relapse transitions, kept in every cohort simulation, resulted in background yearly smoking status changes, both with and without interventions. As compared to current smokers, ex-smokers had a lower disease progression (see supporting information. Table S2), and a lower probability of death (see supporting information Table S3, n.b. a lower probability of exacerbations for ex-smokers was also explored in the sensitivity analysis).

### Disease progression parameter values: cost and health utilities

Costs were based on data from Jansson et al. [22] and included direct and indirect costs. As the study by Jansson et al. was based on the British Thoracic Society classification of COPD severity, these costs were adapted to the GOLD classification. Moreover, as the initial estimates by Jansson et al. were given in 2002 Swedish Crowns, costs were discounted (3.5% rate), converted into 2010 pounds Sterling, and corrected with the Big Mac index [23].

Health utilities were expressed in QALY and based on the estimates reported by Borg et al and Cataluna et al. [18], [24] for COPD patients: respectively 0.8971, 0.7511, 0.7481 and 0.5493 QALY for stable (i.e. exacerbation-free during a one-year iteration) GOLD1, GOLD2, GOLD3 and GOLD4 patients; respectively 0.8951, 0.7364, 0.7261 and 0.5357 QALY, for unstable patients.

### Sensitivity analysis

Various scenarios were examined in order to explore the impact of varying the model parameter values on the simulation outputs (costs, QALY and ICER). In particular, we explored the hypothesis of smoking cessation being due to a smoking cessation programme, allowing the cost and quit rate of such a programme to vary. The remaining parameters that were also allowed to vary were the transition rate from one severity stage to the next, mortality and exacerbation rates, management costs, discounting rates and smoking cessation rates.

## Results

When considering cohorts combining all severity stages at cohort initiation, the simulations estimated that COPD patients remaining continuous smokers until their end of life had a mean remaining lifespan of 15.60 (mean) LY corresponding to 8.47 QALY. Compared to these patients, sustained quitters were estimated to gain 2.73 LY and 1.225 QALY (Table 2). Considering patient's lifespan, the additional disease-related cost of continuous smoking versus sustained abstinence was 1661 £ per patient. Health gains associated with sustained abstinence decreased with disease severity status at cohort initiation, from 3.17 to 1.92 LY and from 1.43 to 0.657 QALY for GOLD1 and GOLD4 patients, respectively (Table 2). The simulations estimated that during a 10-year period, the specific burden of continuous smoking (as compared to sustained abstinence) in the current 1400000 English COPD smokers resulted in total disease-related costs of 1657 M£ together with losses of 452516 QALY.

**Table 2:** The burden of continuous smoking in COPD smoking patients.

Stage of the cohort members at cohort initiation	Mean estimate*						
	Cost† (£) per patient		Life-years per patient		QALY per patient		ICER (£/QALY)
	Continuous smokers	Sustained quitters‡	Continuous smokers	Sustained quitters‡	Continuous smokers	Sustained quitters‡	
All stages combined	27834	−1661	15.60	2.73	8.471	1.225	−1356
GOLD1	12196	−2967	19.96	3.17	11.426	1.434	−2070
GOLD2	30810	−3070	14.31	2.67	7.495	1.183	−2594
GOLD3	47021	3588	10.34	2.02	5.348	0.969	3703
GOLD4	72654	11530	9.83	1.92	4.142	0.657	17546

COPD, chronic obstructive pulmonary disease; QALY, quality adjusted life-years; ICER, incremental cost-effectiveness ratio: additional cost (positive values) or savings (negative values) per unit of QALY gained.

The average age in the initial cohorts “All Stages combined”, GOLD 1, GOLD 2, GOLD 3 and GOLD 4, was 60, 57, 60.5, 64.6 and 64.6, respectively.

*Cumulative value at the horizon of a patient's remaining lifetime.

† Direct costs accounted for 40% of the total costs shown in the Table.

‡ Sustained quitter values are reported as incremental values compared to continuous smoker. A positive number denotes an increase of sustained quitter value above continuous smoker value and a negative number a decrease of value.

All further results concern simulations where relapse and quit events were allowed (see methods and Table 1). When considering cohorts combining all stages, smoking cessation by a patient at cohort entry resulted in health gains of 1.27 LY and 0.68 QALY (Table 3) at the horizon of his remaining lifetime. In addition, cost savings amounted to 1824 £ per patient over his/her remaining lifetime, with a corresponding ICER of −2686 £/QALY. The simulation outputs (life expectancy, QALY and cost) varied according to a given patient's initial COPD stage (Table 3). Overall, the health gains associated with smoking cessation at cohort entry decreased as baseline COPD severity increased (from 0.72 QALY for patients initially in stage GOLD1, to 0.69, 0.64, and 0.42 QALY for patients initially in stages GOLD2, GOLD3 and GOLD4, respectively). We observed savings for patients initially in stage GOLD1 or GOLD2, with the most favourable ICER observed for GOLD2 patients: −4624 £/QALY. GOLD3 and GOLD4 were associated with costs with the less favourable ICER of 17546 £/QALY observed for patients initially in stage GOLD4, a value near the NICE threshold (20000 £/QALY) for declaring an intervention worthwhile (i.e. cost-effective).

**Table 3:** Cost-effectiveness of smoking cessation (intervention) for a COPD smoking patient.

Stage of the cohort members at cohort initiation	Mean estimate*						
	Cost† (£) per patient		Life-years per patient		QALY per patient		ICER (£/QALY)
	No intervention	Intervention‡	No intervention	Intervention‡	No intervention	Intervention‡	
All stages combined	28013	−1824	16.51	1.27	8.810	0.679	−2686
GOLD1	11612	−2004	21.12	1.29	11.869	0.721	−2779
GOLD2	31031	−3171	15.14	1.33	7.804	0.686	−4624
GOLD3	48422	1366	10.87	1.16	5.548	0.641	2133
GOLD4	75222	7349	10.33	1.11	4.288	0.419	17546

COPD, chronic obstructive pulmonary disease; QALY, quality adjusted life-years; ICER, incremental cost-effectiveness ratio: additional cost (positive values) or savings (negative values) per unit of QALY gained.

The average age in the initial cohorts “All Stages combined”, GOLD 1, GOLD 2, GOLD 3 and GOLD 4, was 60, 57, 60.5, 64.6 and 64.6, respectively.

*Cumulative value at the horizon of a patient's remaining lifetime.

† Direct costs accounted for 40% of the total costs shown in the Table.

‡ Intervention (i.e. smoking cessation at cohort initiation) values are reported as incremental values compared to no intervention. A positive number denotes an increase of intervention value above no intervention value and a negative number a decrease of intervention value from no intervention value.

**Figure 2:**
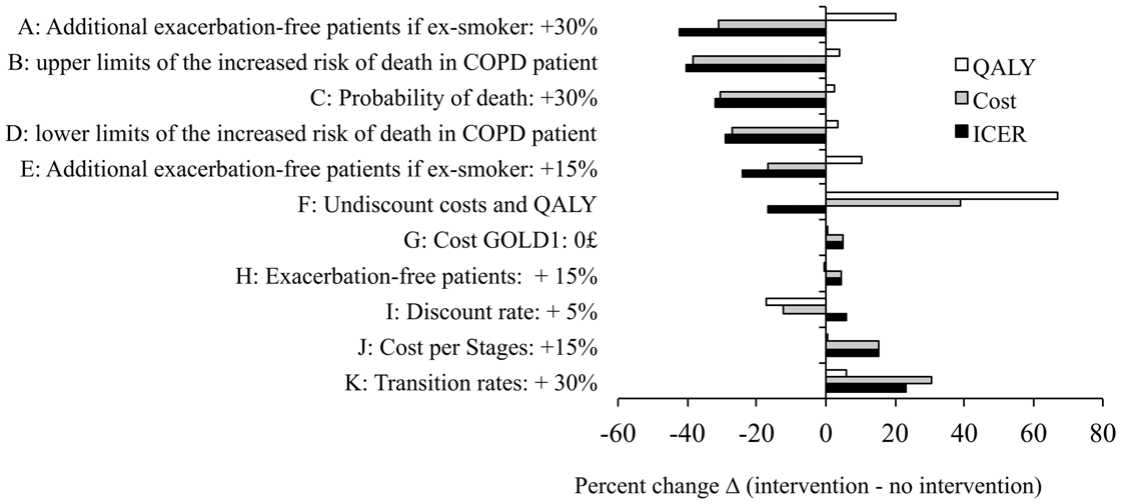
Sensitivity analysis. In each of the A to K explored scenarios (top to bottom), the value of a key parameter was changed as compared to the reference case scenario. For each of these scenarios, the figure indicates how simulation outputs (i.e. difference Δ between intervention and non intervention in terms of QALY, Cost, ICER) change, as compared to the simulation outputs of the reference scenario (reference case for which ΔQALY, ΔCost, ΔICER were 0.679 QALY, −1824 £ and −2686 £/QALY, respectively, see Table 1 for parameter values and Table 3 for more detailed simulation outputs). For example, in scenario A, ΔQALY, ΔCost, ΔICER were 0.817 QALY, −1262 £ and −1544 £/QALY, therefore representing respectively a 20% ((0,817−0,679)/0,679), a −31%, and a −42% change, as compared to the reference case scenario. Scenarios A to K correspond to the following modifications of parameter values as compared to those used in the reference case: A, the proportion of exacerbation-free patients among ex-smokers was raised by 30%; B, the increased risks of death in COPD patients (as compared to individuals of the standard population were set to the upper limits reported by Mannino et al [21]; C, the probability of death was increased by 30%; D, the increased risks of death in COPD patients (as compared to individuals of the standard population) were set to the lower limit reported by Mannino et al [21]; E, the proportion of exacerbation-free patients among ex-smokers was raised by 15%; F, health utilities and costs were not discounted; G, no disease management costs for GOLD1 patients; H, the proportion of exacerbation-free patients was raised by 15%; I, health utilities and costs were discounted at the rate of 5%; J, disease management costs increased by 15% for each severity stage; K, the transition rate from one stage to the next was increased by 30%.

Sensitivity analyses were then used to explore how the results were affected when the parameter values were varied (Table 4 and Figure 2). Considering a cohort of COPD smokers combining all stages at cohort initiation, all simulations corresponding to the reference case scenario showed that a smoking cessation intervention would lead to cost savings, whatever its cost (Table 4). When the percentage of quitters after the smoking cessation intervention was varied, only 3 scenarios resulted in ICERs>5000 £/QALY. The least favourable situation, 14965 £/QALY, a figure still below the NICE threshold, corresponded to an intervention having a cost of 1000£ and an initial rate of 1/12 quitters after the smoking cessation intervention. When the results were analysed according to initial severity, smoking cessation appeared especially favourable for GOLD2 patients. Nevertheless, even for GOLD3 patients, only one hypothesis corresponding to an intervention having a cost of 1000£ and an initial rate of 1/12 quitters after the smoking cessation intervention exceeded the threshold of a “worthwhile” ICERs. In contrast, ICERs derived from simulations of patients initially at stage GOLD4 often exceeded 20000 £/QALY, and the most favourable scenarios were near this limit.

**Table 4:** Cost-effectiveness of smoking cessation according to the abstinence rate.

*Stage of the cohort members at cohort initiation* (initial abstinence rate)	COPD cost for the remaining lifetime (£)	Remaining life-years	Remaining quality adjusted life-years (QALY)	ICER (£/QALY) according to the cost (£) of a smoking cessation programme				
				0 £	100 £	200 £	500 £	1000 £
*All_Stages*								
No intervention (0%)	28013	16.51	8.810					
Reference case (100%)	−1824	1.27	0.679	−2686	−2539	−2392	−1950	−1214
1/3 (33%)	−606	0.43	0.228	−2659	−2219	−1781	−465	1728
1/6 (17%)	−300	0.21	0.114	−2643	−1754	−877	1754	6140
1/12 (8%)	−147	0.11	0.057	−2599	−825	930	6193	14965
*GOLD1*								
No intervention (0%)	11612	21.12	11.869					
Reference case (100%)	−2004	1.29	0.721	−2779	−2641	−2502	−2086	−1393
1/3 (33%)	−677	0.43	0.241	−2808	−2394	−1979	−734	1340
1/6 (17%)	−337	0.22	0.122	−2770	−1943	−1123	1336	5434
1/12 (8%)	−175	0.11	0.061	−2864	−1230	410	5328	13525
*GOLD2*								
No intervention (0%)	31031	15.14	7.804					
Reference case (100%)	−3171	1.33	0.686	−4624	−4477	−4331	−3894	−3165
1/3 (33%)	−1062	0.45	0.230	−4626	−4183	−3748	−2443	−270
1/6 (17%)	−523	0.22	0.114	−4568	−3711	−2833	−202	4184
1/12 (8%)	−258	0.11	0.057	−4516	−2772	−1018	4246	13018
*GOLD3*								
No intervention (0%)	48422	10.87	5.548					
Reference case (100%)	1366	1.16	0.641	2133	2287	2443	2911	3691
1/3 (33%)	438	0.38	0.214	2044	2514	2981	4383	6720
1/6 (17%)	224	0.19	0.107	2093	3028	3963	6766	11439
1/12 (8%)	106	0.09	0.053	2013	3887	5774	11434	20868
*GOLD4*								
No intervention (0%)	75222	10.33	4.288					
Reference case (100%)	7349	1.11	0.419	17546	17778	18017	18733	19926
1/3 (33%)	2454	0.37	0.140	17547	18243	18957	21100	24671
1/6 (17%)	1219	0.18	0.069	17548	19116	20565	24913	32159
1/12 (8%)	605	0.09	0.034	17547	20735	23676	32500	47206

COPD, chronic obstructive pulmonary disease; QALY, quality adjusted life-years; ICER, incremental cost-effectiveness ratio: additional cost (positive values) or savings (negative values) per unit of QALY gained.

All values except those corresponding to no intervention (cohorts in which all patients smoke at simulation initiation) represent incremental costs, incremental health outcomes, or incremental cost-effectiveness, as compared to no intervention.

The results were sensitive to the transition rates from one severity stage to the next (Figure 2, scenario K), and to an increase in the mortality rates (Figure 2, scenarios B, C and D). The model was not very sensitive to an exacerbation-free rate 15% higher than that of the reference case (Figure 2, scenario H). In contrast, when we postulated that ex-smokers experienced fewer exacerbations than smokers (the two explored scenarios hypothesized that on average, the probability of an exacerbation-free year was respectively 15% or 30% higher in ex-smokers than in current smokers, see Figure 2 scenarios A and E), monetary and healthcare gains were observed, with the ICER rising by 42% in the most favourable scenario. The model was not very sensitive to variations in disease management costs: hypothesizing a 15% additional cost for each severity stage (Figure 2, scenario G) or no cost at all for GOLD1 patients (Figure 2, scenario J) resulted in modest changes in the ICER estimates. When health utilities and costs were not discounted (Figure 2, scenario F) and or when a discounting rate of 5% (Figure 2, scenario I) was applied as recommended in international guidelines [12], the changes in the corresponding ICERs were also modest. Overall, it's worth to mention that all the scenarios shown in Figure 2 remained yielding savings in terms of the ICERs, as the percentage change relative to the reference case (−2686 £/QALY) was always below 100%.

## Discussion

The proposed model allowed to estimate what represents the specific burden of continuous smoking for a COPD smoker (as compared to a COPD smoker becoming a sustained quitter): 2.73 LY with corresponding 1.22 QALY lost, and additional 1661 £ disease-related costs (Table 2). One might consider these patient's level differences between sustained smoking versus sustained abstinence as modest. However, LY estimate is close to that of Doll et al. who studied a population of smoking male British doctors aged 60 years (the mean age in our COPD simulated cohorts was 60 years) and found that sustained smoking cessation added 3 years of life expectancy [25]. Moreover, the 1400000 English COPD smokers [16] represent 4.6% of the general population of the same generation [7], and the gains for society at a national level are substantial (see results).

When relapse and quit events were allowed as observed in everyday life, the simulation results indicated that a randomly selected COPD patient who quits smoking might expect a gain of 1.27-year in his/her remaining lifespan with corresponding 0.68 QALY. All in all, these results indicate that sustained abstinence after smoking cessation roughly doubles the health gains when compared to smoking cessation with relapses. Smoking cessation by English COPD patients would also reduce COPD management costs.

The study also examines in detail the cost-effectiveness of smoking cessation according to disease severity and the results have practical consequences. First, whatever the disease stage considered, smoking cessation led to modest healthcare gains considering the remaining life of a COPD patient (Table 3). Such a knowledge may be considered as a useful contribution for supporting upstream public health policies in the domain of smoking cessation programmes devoted to younger populations, in order to decrease the number of COPD future cases. Second, the study also suggests the development of smoking cessation programmes targeted to specific populations of COPD patients: smoking cessation would result in worthwhile ICERs for patients with both mild and severe COPD in most cases, even when hypothesizing expensive programmes and low rates of quitters (Table 4). For example, a smoking cessation programme targeting patients progressing from GOLD1 to GOLD2 appears especially attractive: an intervention targeted to GOLD2 patients, costing 200 £ and having a success rate of only 8% (lower than the 12.3% success rate for a COPD patient reported in a review [9], [10]), would yield an ICER of −1018 £/QALY. We examined how the results were affected by varying the smoking cessation rate (Table 4), a parameter associated with a substantial uncertainty. In the general population, 9 to 12 weeks of pharmacological therapy might cost between £81.56 and £165.66, and yield a 12-month abstinence rate of between 3% and 23% [26], [27]. A review [28] of smoking cessation interventions in COPD patients (general COPD population, general practitioners, hospital inpatients) reported 12-month abstinence rates ranging from 4.5% [29] to 34.5% [30]. COPD patients are more depressed and prone to relapse [31]. They might find it more difficult to quit than smokers in the general population, but current and future social and medical pressures might increase the quit rates. Smoking cessation programmes specifically dedicated to COPD patients might cost more than those targeted to a general population of smokers, but our study explored a large range of costs for such programmes, and a favourable ICER was obtained with most of the postulated smoking cessation rates and intervention costs.

Three medico-economic studies [9], [10], [32] focused on the impact of smoking on Dutch COPD patients. The first study [32] estimated the impact of changes in demographics and tobacco consumption on COPD morbidity, mortality and costs during a 20-year period, and indicated that 90% of disease costs were accounted for by COPD patients who were current or ex-smokers. The two other studies [9], [10] analyzed the consequences of various smoking cessation programmes on COPD patients during a 25-year period. In particular, a programme involving intensive counseling plus pharmacotherapy dominated the other interventions [9]. The underlying models [10], [32] considered COPD included predictions of the impact of incident COPD during the study period. Such a perspective has the advantage of cumulating the whole burden (associated with current and future patients) within a fixed horizon time. At the opposite, a disadvantage of such a perspective is that incidence data being censored, the estimates in patient-years cannot be converted to estimates per patient. In contrast, our study based on current COPD data for England, is patient-centred. The proposed simulations followed all cohort members until death and detail the cost-effectiveness impact of smoking cessation according to disease severity, examining simultaneously various potential costs of smoking cessation programmes and various potential rates of associated quit rates. To our knowledge, the patient estimates reported in the present study have never yet been reported. Moreover, the material provided in the present study may also be viewed as a proposed accessible tool for exploring in details the medico-economic impact of smoking cessation in COPD patients, assuming a wide range of hypotheses.

The results of this study are limited by uncertainties on some of the parameter values. For example, the transition rates between severity stages [17] were not specific to the English population of COPD patients, and mortality rates by age and smoking status among English COPD patients have not been reported. Nevertheless, varying the transition rates and the mortality rates (COPD-related mortality is thought to be underestimated [33], [34]) did not markedly affect the ICERs (Figure 2). Another problem concerns exacerbations, for which there is no agreed definition [35]. Our results indicate that smoking cessation remains cost-effective even when the proportion of exacerbation-free patients increases or when ex-smokers experience fewer exacerbations (Figure 2). Other uncertainties concern the costs of the disease. First, the cost of COPD has not been estimated in the English general population, and our model parameter values were thus based on Swedish data. Nevertheless, our results were not very sensitive to variations in such costs. This may be due to the initially high proportion of patients in stage GOLD1, which is associated with negligible costs but adding 15% to the cost of each severity stage resulted in only moderate changes in the simulation outputs (Figure 2). Second, neither the relative frequency of comorbidities in COPD smokers and ex-smokers have been reported, neither their associated specific cost. Since such costs were therefore not taken into account in the simulations whereas the corresponding health outcomes were (health utilities and all-cause death rates used in the simulations include comorbidities), the cost-effectiveness estimates issued from the simulations should be considered as a lower limit, the value of the increase in these estimates related to taking into account the costs of comorbidities (likely more important in COPD smokers than in COPD ex-smokers) remaining to be studied. The dependency of the results on the chosen values of certain parameters is also useful for guiding design and data collection in future studies, in order to obtain estimates of better quality. More precise estimates of transition rates between severity stages, mortality and exacerbation rates, patient age distribution and background changes in smoking status would help to guide new strategies designed to improve COPD patient management. Most of all, even if sensitivity analysis did not outline any critical inappropriate parameter value used in our model, since the health conditions and medical treatment choices improve every year, more recent parameter values than those used in our study would improve the quality of the model outputs.

In conclusion, this study indicates that smoking cessation by English COPD patients would be associated with gains in life expectancy and quality of life, and with a reduction in disease-related costs. These gains might be considered as modest for the individual patient but are substantial from society's standpoint. Smoking cessation interventions focusing on GOLD1 and GOLD2 patients are expected to be particularly beneficial, even if devoted programmes, likely more expensive than interventions in other populations, are considered.

## Supporting Information

Table S1 Initial age distributions (% of patients) of English COPD smoking patients according to severity.
(DOC)

Table S2 Transition probabilities from a severity stage to the next.
(DOC)

Table S3 Probability of Death of a COPD patient according to age, smoking status, and severity.
(DOC)
