# Supplementary material for: Simulation-Based Estimates of Effectiveness and Cost-Effectiveness of Smoking Cessation in Patients with Chronic Obstructive Pulmonary Disease
Source: PLoS One. 2011 Sep 14;6(9):e24870. doi: 10.1371/journal.pone.0024870 (PMC3173494; doi:10.1371/journal.pone.0024870)
Supplement: Table S1 — Initial age distributions (% of patients) of English COPD smoking patients according to severity. (DOC) [file pone.0024870.s001.doc]

**Table S1. Initial age distributions (% of patients) of English COPD smoking patients according to severity stage**.

| Age | COPD severity stage | | | | | |
| --- | --- | --- | --- | --- | --- | --- |
|  |  | GOLD 1 |  | GOLD 2 |  | GOLD 3-4 |
| 40 |  | 2.04 |  | 1.09 |  | 0.36 |
| 41 |  | 2.08 |  | 1.12 |  | 0.36 |
| 42 |  | 2.07 |  | 1.11 |  | 0.37 |
| 43 |  | 2.09 |  | 1.12 |  | 0.37 |
| 44 |  | 2.06 |  | 1.10 |  | 0.36 |
| 45 |  | 2.78 |  | 2.18 |  | 1.01 |
| 46 |  | 2.70 |  | 2.13 |  | 0.99 |
| 47 |  | 2.64 |  | 2.07 |  | 0.96 |
| 48 |  | 2.54 |  | 1.99 |  | 0.92 |
| 49 |  | 2.49 |  | 1.95 |  | 0.91 |
| 50 |  | 2.45 |  | 1.92 |  | 0.89 |
| 51 |  | 2.35 |  | 1.85 |  | 0.86 |
| 52 |  | 2.27 |  | 1.78 |  | 0.83 |
| 53 |  | 2.21 |  | 1.73 |  | 0.80 |
| 54 |  | 2.22 |  | 1.74 |  | 0.81 |
| 55 |  | 2.30 |  | 2.27 |  | 2.59 |
| 56 |  | 2.27 |  | 2.26 |  | 2.58 |
| 57 |  | 2.29 |  | 2.26 |  | 2.58 |
| 58 |  | 2.34 |  | 2.32 |  | 2.65 |
| 59 |  | 2.41 |  | 2.39 |  | 2.72 |
| 60 |  | 2.58 |  | 2.55 |  | 2.92 |
| 61 |  | 2.70 |  | 2.66 |  | 3.04 |
| 62 |  | 2.18 |  | 2.16 |  | 2.47 |
| 63 |  | 2.12 |  | 2.10 |  | 2.39 |
| 64 |  | 2.06 |  | 2.04 |  | 2.33 |
| 65 |  | 2.72 |  | 3.39 |  | 4.09 |
| 66 |  | 2.44 |  | 3.04 |  | 3.68 |
| 67 |  | 2.27 |  | 2.84 |  | 3.42 |
| 68 |  | 2.35 |  | 2.93 |  | 3.54 |
| 69 |  | 2.32 |  | 2.90 |  | 3.51 |
| 70 |  | 2.28 |  | 2.84 |  | 3.43 |
| 71 |  | 2.20 |  | 2.75 |  | 3.32 |
| 72 |  | 2.12 |  | 2.64 |  | 3.19 |
| 73 |  | 2.02 |  | 2.53 |  | 3.05 |
| 74 |  | 1.91 |  | 2.38 |  | 2.88 |
| 75 |  | 1.86 |  | 2.32 |  | 2.80 |
| 76 |  | 1.83 |  | 2.28 |  | 2.75 |
| 77 |  | 1.77 |  | 2.22 |  | 2.68 |
| 78 |  | 1.69 |  | 2.10 |  | 2.53 |
| 79 |  | 1.57 |  | 1.95 |  | 2.37 |
| 80 |  | 1.44 |  | 1.81 |  | 2.18 |
| 81 |  | 1.38 |  | 1.72 |  | 2.08 |
| 82 |  | 1.30 |  | 1.62 |  | 1.95 |
| 83 |  | 1.18 |  | 1.47 |  | 1.78 |
| 84 |  | 1.08 |  | 1.35 |  | 1.63 |
| 85 |  | 0.99 |  | 1.23 |  | 1.48 |
| 86 |  | 0.92 |  | 1.15 |  | 1.39 |
| 87 |  | 0.87 |  | 1.08 |  | 1.31 |
| 88 |  | 0.72 |  | 0.91 |  | 1.09 |
| 89 |  | 0.53 |  | 0.66 |  | 0.80 |
