# Supplementary material for: Simulation-Based Estimates of Effectiveness and Cost-Effectiveness of Smoking Cessation in Patients with Chronic Obstructive Pulmonary Disease
Source: PLoS One. 2011 Sep 14;6(9):e24870. doi: 10.1371/journal.pone.0024870 (PMC3173494; doi:10.1371/journal.pone.0024870)
Supplement: Table S2 — Transition probabilities from a severity stage to the next. (DOC) [file pone.0024870.s002.doc]

**Table S2.** Transition probabilities from a severity stage to the next.

| Age |  | Ex-Smoker | | | | |  | Current Smoker | | | | |
| --- | --- | --- | --- | --- | --- | --- | --- | --- | --- | --- | --- | --- |
|  |  | GOLD1 to GOLD2 |  | GOLD2 to GOLD3 |  | GOLD3 to GOLD4 |  | GOLD1 to GOLD2 |  | GOLD2 to GOLD3 |  | GOLD3 to GOLD4 |
| 40 |  | 0.01733 |  | 0.03867 |  | 0.03412 |  | 0.02763 |  | 0.06226 |  | 0.05217 |
| 41 |  | 0.01762 |  | 0.03930 |  | 0.03467 |  | 0.02807 |  | 0.06326 |  | 0.05301 |
| 42 |  | 0.01790 |  | 0.03992 |  | 0.03522 |  | 0.02851 |  | 0.06427 |  | 0.05385 |
| 43 |  | 0.01818 |  | 0.04054 |  | 0.03577 |  | 0.02894 |  | 0.06527 |  | 0.05469 |
| 44 |  | 0.01846 |  | 0.04116 |  | 0.03632 |  | 0.02938 |  | 0.06627 |  | 0.05553 |
| 45 |  | 0.01874 |  | 0.04178 |  | 0.03687 |  | 0.02982 |  | 0.06727 |  | 0.05637 |
| 46 |  | 0.01902 |  | 0.04240 |  | 0.03741 |  | 0.03025 |  | 0.06828 |  | 0.05721 |
| 47 |  | 0.01930 |  | 0.04302 |  | 0.03796 |  | 0.03069 |  | 0.06928 |  | 0.05805 |
| 48 |  | 0.01969 |  | 0.04388 |  | 0.03872 |  | 0.03130 |  | 0.07067 |  | 0.05921 |
| 49 |  | 0.02008 |  | 0.04474 |  | 0.03948 |  | 0.03191 |  | 0.07206 |  | 0.06038 |
| 50 |  | 0.02047 |  | 0.04560 |  | 0.04023 |  | 0.03252 |  | 0.07345 |  | 0.06154 |
| 51 |  | 0.02087 |  | 0.04645 |  | 0.04099 |  | 0.03312 |  | 0.07484 |  | 0.06271 |
| 52 |  | 0.02126 |  | 0.04731 |  | 0.04174 |  | 0.03373 |  | 0.07624 |  | 0.06387 |
| 53 |  | 0.02188 |  | 0.04871 |  | 0.04298 |  | 0.03474 |  | 0.07848 |  | 0.06575 |
| 54 |  | 0.02250 |  | 0.05012 |  | 0.04422 |  | 0.03574 |  | 0.08072 |  | 0.06763 |
| 55 |  | 0.02312 |  | 0.05152 |  | 0.04546 |  | 0.03674 |  | 0.08296 |  | 0.06951 |
| 56 |  | 0.02374 |  | 0.05292 |  | 0.04670 |  | 0.03774 |  | 0.08520 |  | 0.07139 |
| 57 |  | 0.02436 |  | 0.05432 |  | 0.04793 |  | 0.03875 |  | 0.08745 |  | 0.07327 |
| 58 |  | 0.02502 |  | 0.05556 |  | 0.04902 |  | 0.03979 |  | 0.08942 |  | 0.07492 |
| 59 |  | 0.02568 |  | 0.05679 |  | 0.05011 |  | 0.04084 |  | 0.09140 |  | 0.07658 |
| 60 |  | 0.02633 |  | 0.05803 |  | 0.05120 |  | 0.04188 |  | 0.09338 |  | 0.07823 |
| 61 |  | 0.02699 |  | 0.05926 |  | 0.05229 |  | 0.04293 |  | 0.09535 |  | 0.07989 |
| 62 |  | 0.02765 |  | 0.06049 |  | 0.05338 |  | 0.04397 |  | 0.09733 |  | 0.08155 |
| 63 |  | 0.02790 |  | 0.06104 |  | 0.05386 |  | 0.04438 |  | 0.09822 |  | 0.08229 |
| 64 |  | 0.02816 |  | 0.06159 |  | 0.05434 |  | 0.04478 |  | 0.09912 |  | 0.08304 |
| 65 |  | 0.02841 |  | 0.06213 |  | 0.05482 |  | 0.04519 |  | 0.10001 |  | 0.08379 |
| 66 |  | 0.02866 |  | 0.06268 |  | 0.05530 |  | 0.04560 |  | 0.10091 |  | 0.08454 |
| 67 |  | 0.02891 |  | 0.06322 |  | 0.05579 |  | 0.04600 |  | 0.10180 |  | 0.08529 |
| 68 |  | 0.02912 |  | 0.06367 |  | 0.05618 |  | 0.04633 |  | 0.10252 |  | 0.08589 |
| 69 |  | 0.02934 |  | 0.06412 |  | 0.05658 |  | 0.04665 |  | 0.10324 |  | 0.08650 |
| 70 |  | 0.02956 |  | 0.06457 |  | 0.05698 |  | 0.04698 |  | 0.10396 |  | 0.08710 |
| 71 |  | 0.02977 |  | 0.06502 |  | 0.05737 |  | 0.04730 |  | 0.10468 |  | 0.08770 |
| 72 |  | 0.02999 |  | 0.06547 |  | 0.05777 |  | 0.04763 |  | 0.10540 |  | 0.08831 |
| 73 |  | 0.03005 |  | 0.06561 |  | 0.05789 |  | 0.04773 |  | 0.10562 |  | 0.08849 |
| 74 |  | 0.03010 |  | 0.06575 |  | 0.05801 |  | 0.04783 |  | 0.10584 |  | 0.08868 |
| 75 |  | 0.03016 |  | 0.06589 |  | 0.05814 |  | 0.04793 |  | 0.10607 |  | 0.08887 |
| 76 |  | 0.03021 |  | 0.06603 |  | 0.05826 |  | 0.04803 |  | 0.10629 |  | 0.08905 |
| 77 |  | 0.03027 |  | 0.06617 |  | 0.05838 |  | 0.04813 |  | 0.10651 |  | 0.08924 |
| 78 |  | 0.03036 |  | 0.06638 |  | 0.05857 |  | 0.04828 |  | 0.10686 |  | 0.08953 |
| 79 |  | 0.03046 |  | 0.06659 |  | 0.05876 |  | 0.04844 |  | 0.10720 |  | 0.08982 |
| 80 |  | 0.03056 |  | 0.06681 |  | 0.05895 |  | 0.04859 |  | 0.10755 |  | 0.09011 |
| 81 |  | 0.03066 |  | 0.06702 |  | 0.05914 |  | 0.04875 |  | 0.10789 |  | 0.09040 |
| 82 |  | 0.03075 |  | 0.06724 |  | 0.05933 |  | 0.04890 |  | 0.10824 |  | 0.09069 |
| 83 |  | 0.03107 |  | 0.06792 |  | 0.05993 |  | 0.04940 |  | 0.10935 |  | 0.09161 |
| 84 |  | 0.03138 |  | 0.06861 |  | 0.06054 |  | 0.04990 |  | 0.11045 |  | 0.09254 |
| 85 |  | 0.03170 |  | 0.06930 |  | 0.06114 |  | 0.05040 |  | 0.11156 |  | 0.09347 |
| 86 |  | 0.03201 |  | 0.06998 |  | 0.06175 |  | 0.05091 |  | 0.11266 |  | 0.09439 |
| 87 |  | 0.03233 |  | 0.07067 |  | 0.06236 |  | 0.05141 |  | 0.11377 |  | 0.09532 |
| 88 |  | 0.03264 |  | 0.07136 |  | 0.06296 |  | 0.05191 |  | 0.11487 |  | 0.09624 |
| 89 |  | 0.03296 |  | 0.07204 |  | 0.06357 |  | 0.05241 |  | 0.11598 |  | 0.09717 |
| 90 |  | 0.03327 |  | 0.07273 |  | 0.06417 |  | 0.05291 |  | 0.11708 |  | 0.09810 |
| 91 |  | 0.03358 |  | 0.07342 |  | 0.06478 |  | 0.05341 |  | 0.11819 |  | 0.09902 |
| 92 |  | 0.03390 |  | 0.07410 |  | 0.06538 |  | 0.05391 |  | 0.11929 |  | 0.09995 |
| 93 |  | 0.03421 |  | 0.07479 |  | 0.06599 |  | 0.05441 |  | 0.12040 |  | 0.10088 |
| 94 |  | 0.03453 |  | 0.07547 |  | 0.06659 |  | 0.05492 |  | 0.12150 |  | 0.10180 |
| 95 |  | 0.03484 |  | 0.07616 |  | 0.06720 |  | 0.05542 |  | 0.12261 |  | 0.10273 |
| 96 |  | 0.03516 |  | 0.07685 |  | 0.06781 |  | 0.05592 |  | 0.12372 |  | 0.10365 |
| 97 |  | 0.03547 |  | 0.07753 |  | 0.06841 |  | 0.05642 |  | 0.12482 |  | 0.10458 |
| 98 |  | 0.03579 |  | 0.07822 |  | 0.06902 |  | 0.05692 |  | 0.12593 |  | 0.10551 |
| 99-110 |  | 0.03610 |  | 0.07891 |  | 0.06962 |  | 0.05742 |  | 0.12703 |  | 0.10643 |

Nielsen et al [1] reported transition probabilities from GOLD1 to GOLD2 and from GOLD2 to GOLD3 (but not from GOLD3 to GOLD4) by age and smoking status (current smokers versus non-smokers/ex-smokers). To set the transition probability from GOLD3 to GOLD4, we used the data reported by Hoogendorn et al [2] who reported the transition probabilities from GOLD2 to GOLD3 and from GOLD3 to GOLD4 according to smoking status (but not age): the ratio between these two probabilities was considered to be similar to that of the Framingham cohort [1], thus resulting in a transition probability table by age, severity stage and smoking status.

References

1. Nielsen R, Johannessen A, Benediktsdottir B, Gislason T, Buist AS, et al. (2009) Present and future costs of COPD in Iceland and Norway: results from the BOLD study. Eur Respir J 34: 850-857
2. Hoogendoorn M, Rutten-van Molken MP, Hoogenveen RT, van Genugten ML, Buist AS, et al. (2005) A dynamic population model of disease progression in COPD. Eur Respir J 26: 223-233.
