# Supplementary material for: Simulation-Based Estimates of Effectiveness and Cost-Effectiveness of Smoking Cessation in Patients with Chronic Obstructive Pulmonary Disease
Source: PLoS One. 2011 Sep 14;6(9):e24870. doi: 10.1371/journal.pone.0024870 (PMC3173494; doi:10.1371/journal.pone.0024870)
Supplement: Table S3 — Probability of Death of a COPD patient according to age, smoking status, and severity. (DOC) [file pone.0024870.s003.doc]

**Table S3.** Probability of Death of a COPD patient according to age, smoking status, and severity.

| Age |  | Ex-smokers | | | | | | | | | | |  | Current smokers | | | | | | | | | | |
| --- | --- | --- | --- | --- | --- | --- | --- | --- | --- | --- | --- | --- | --- | --- | --- | --- | --- | --- | --- | --- | --- | --- | --- | --- |
|  |  | GOLD1 | | |  | GOLD2 | | |  | GOLD3 or GOLD4 | | |  | GOLD 1 | | |  | GOLD 2 | | |  | GOLD 3 or GOLD4 | | |
|  |  | E- |  | E+ |  | E- |  | E+ |  | E- |  | E+ |  | E- |  | E+ |  | E- |  | E+ |  | E- |  | E+ |
| 40-44 |  | 0.00148 |  | 0.00237 |  | 0.00237 |  | 0.00312 |  | 0.00282 |  | 0.00668 |  | 0.00210 |  | 0.00337 |  | 0.00337 |  | 0.00442 |  | 0.00400 |  | 0.00947 |
| 45-49 |  | 0.00231 |  | 0.00369 |  | 0.00369 |  | 0.00485 |  | 0.00439 |  | 0.01039 |  | 0.00327 |  | 0.00524 |  | 0.00524 |  | 0.00687 |  | 0.00622 |  | 0.01473 |
| 50-54 |  | 0.00365 |  | 0.00583 |  | 0.00583 |  | 0.00766 |  | 0.00693 |  | 0.01641 |  | 0.00517 |  | 0.00827 |  | 0.00827 |  | 0.01086 |  | 0.00982 |  | 0.02327 |
| 55-59 |  | 0.00556 |  | 0.00889 |  | 0.00889 |  | 0.01167 |  | 0.01056 |  | 0.02500 |  | 0.00788 |  | 0.01261 |  | 0.01261 |  | 0.01655 |  | 0.01497 |  | 0.03546 |
| 60-64 |  | 0.00862 |  | 0.01378 |  | 0.01378 |  | 0.01809 |  | 0.01637 |  | 0.03877 |  | 0.01222 |  | 0.01955 |  | 0.01955 |  | 0.02566 |  | 0.02322 |  | 0.05499 |
| 65-69 |  | 0.01366 |  | 0.02186 |  | 0.02186 |  | 0.02869 |  | 0.02596 |  | 0.06149 |  | 0.01938 |  | 0.03101 |  | 0.03101 |  | 0.04070 |  | 0.03682 |  | 0.08721 |
| 70-74 |  | 0.02219 |  | 0.03551 |  | 0.03551 |  | 0.04661 |  | 0.04217 |  | 0.09987 |  | 0.03148 |  | 0.05036 |  | 0.05036 |  | 0.06610 |  | 0.05981 |  | 0.14165 |
| 75-79 |  | 0.03795 |  | 0.06072 |  | 0.06072 |  | 0.07969 |  | 0.07210 |  | 0.17076 |  | 0.05382 |  | 0.08612 |  | 0.08612 |  | 0.11303 |  | 0.10226 |  | 0.24221 |
| 80-84 |  | 0.06447 |  | 0.10315 |  | 0.10315 |  | 0.13539 |  | 0.12249 |  | 0.29011 |  | 0.09144 |  | 0.14631 |  | 0.14631 |  | 0.19203 |  | 0.17374 |  | 0.41149 |
| 85 |  | 0.09968 |  | 0.15949 |  | 0.15949 |  | 0.20933 |  | 0.18940 |  | 0.44857 |  | 0.14139 |  | 0.22622 |  | 0.22622 |  | 0.29691 |  | 0.26863 |  | 0.63624 |
| 86 |  | 0.13489 |  | 0.21583 |  | 0.21583 |  | 0.28328 |  | 0.25630 |  | 0.60703 |  | 0.19133 |  | 0.30613 |  | 0.30613 |  | 0.40179 |  | 0.36353 |  | 0.86099 |
| 87 |  | 0.17011 |  | 0.27217 |  | 0.27217 |  | 0.35723 |  | 0.32320 |  | 0.76548 |  | 0.24127 |  | 0.38604 |  | 0.38604 |  | 0.50668 |  | 0.45842 |  | 1 |
| 88 |  | 0.20532 |  | 0.32851 |  | 0.32851 |  | 0.43117 |  | 0.39011 |  | 0.92394 |  | 0.29122 |  | 0.46595 |  | 0.46595 |  | 0.61156 |  | 0.55332 |  | 1 |
| 89 |  | 0.24053 |  | 0.38485 |  | 0.38485 |  | 0.50512 |  | 0.45701 |  | 1 |  | 0.34116 |  | 0.54586 |  | 0.54586 |  | 0.71644 |  | 0.64821 |  | 1 |
| 90 |  | 0.27575 |  | 0.44119 |  | 0.44119 |  | 0.57907 |  | 0.52392 |  | 1 |  | 0.39111 |  | 0.62577 |  | 0.62577 |  | 0.82133 |  | 0.74311 |  | 1 |
| 91 |  | 0.31096 |  | 0.49753 |  | 0.49753 |  | 0.65301 |  | 0.59082 |  | 1 |  | 0.44105 |  | 0.70569 |  | 0.70569 |  | 0.92621 |  | 0.83800 |  | 1 |
| 92 |  | 0.34617 |  | 0.55387 |  | 0.55387 |  | 0.72696 |  | 0.65773 |  | 1 |  | 0.49100 |  | 0.78560 |  | 0.78560 |  | 1 |  | 0.93290 |  | 1 |
| 93 |  | 0.38138 |  | 0.61021 |  | 0.61021 |  | 0.80091 |  | 0.72463 |  | 1 |  | 0.54094 |  | 0.86551 |  | 0.86551 |  | 1 |  | 1 |  | 1 |
| 94 |  | 0.41660 |  | 0.66655 |  | 0.66655 |  | 0.87485 |  | 0.79153 |  | 1 |  | 0.59089 |  | 0.94542 |  | 0.94542 |  | 1 |  | 1 |  | 1 |
| 95 |  | 0.45181 |  | 0.72289 |  | 0.72289 |  | 0.94880 |  | 0.85844 |  | 1 |  | 0.64083 |  | 1 |  | 1 |  | 1 |  | 1 |  | 1 |
| 96 |  | 0.48702 |  | 0.77924 |  | 0.77924 |  | 1 |  | 0.92534 |  | 1 |  | 0.69078 |  | 1 |  | 1 |  | 1 |  | 1 |  | 1 |
| 97 |  | 0.52223 |  | 0.83558 |  | 0.83558 |  | 1 |  | 0.99225 |  | 1 |  | 0.74072 |  | 1 |  | 1 |  | 1 |  | 1 |  | 1 |
| 98 |  | 0.55745 |  | 0.89192 |  | 0.89192 |  | 1 |  | 1 |  | 1 |  | 0.79067 |  | 1 |  | 1 |  | 1 |  | 1 |  | 1 |
| 99 |  | 0.59266 |  | 0.94826 |  | 0.94826 |  | 1 |  | 1 |  | 1 |  | 0.84061 |  | 1 |  | 1 |  | 1 |  | 1 |  | 1 |
| 100 |  | 0.62787 |  | 1 |  | 1 |  | 1 |  | 1 |  | 1 |  | 0.89055 |  | 1 |  | 1 |  | 1 |  | 1 |  | 1 |
| 101 |  | 0.66309 |  | 1 |  | 1 |  | 1 |  | 1 |  | 1 |  | 0.94050 |  | 1 |  | 1 |  | 1 |  | 1 |  | 1 |
| 102 |  | 0.69830 |  | 1 |  | 1 |  | 1 |  | 1 |  | 1 |  | 0.99044 |  | 1 |  | 1 |  | 1 |  | 1 |  | 1 |
| 103 |  | 0.73351 |  | 1 |  | 1 |  | 1 |  | 1 |  | 1 |  | 1 |  | 1 |  | 1 |  | 1 |  | 1 |  | 1 |
| 104 |  | 0.76872 |  | 1 |  | 1 |  | 1 |  | 1 |  | 1 |  | 1 |  | 1 |  | 1 |  | 1 |  | 1 |  | 1 |
| 105 |  | 0.80394 |  | 1 |  | 1 |  | 1 |  | 1 |  | 1 |  | 1 |  | 1 |  | 1 |  | 1 |  | 1 |  | 1 |
| 106 |  | 0.83915 |  | 1 |  | 1 |  | 1 |  | 1 |  | 1 |  | 1 |  | 1 |  | 1 |  | 1 |  | 1 |  | 1 |
| 107 |  | 0.87436 |  | 1 |  | 1 |  | 1 |  | 1 |  | 1 |  | 1 |  | 1 |  | 1 |  | 1 |  | 1 |  | 1 |
| 108 |  | 0.90957 |  | 1 |  | 1 |  | 1 |  | 1 |  | 1 |  | 1 |  | 1 |  | 1 |  | 1 |  | 1 |  | 1 |
| 109 |  | 0.94479 |  | 1 |  | 1 |  | 1 |  | 1 |  | 1 |  | 1 |  | 1 |  | 1 |  | 1 |  | 1 |  | 1 |
| 110 |  | 1 |  | 1 |  | 1 |  | 1 |  | 1 |  | 1 |  | 1 |  | 1 |  | 1 |  | 1 |  | 1 |  | 1 |

*Definition of abbreviations:* E- and E+ correspond to exacerbation-free patients and to patients with at least one exacerbation, respectively.

First, based on all-cause mortality data (codes A00-Y89) for the UK general population in 2007, derived from the Eurostat database [1], a table of death probability by age (40 to 110 years) was built. To take into account the excess mortality associated with COPD, we used data from Mannino et al [2], who provide such data for GOLD1, GOLD2 and GOLD3/4 patients, taking exacerbations into account: the relative risks of death were 1.0 (GOLD1), 1.6 (GOLD2) and 1.9 (GOLD3 and GOLD4) for exacerbation-free patients, and 1.6 (GOLD1), 2.1 (GOLD2) and 4.5 (GOLD3 and GOLD4) for patients with exacerbation(s). Finally, the specific mortality table used in our simulations took smoking status into account: based on the report by Ekberg-Aronsson et al [3], the probability of death for COPD ex-smokers and current smokers was considered to be respectively 1.5 and 2.0 times higher than that of never smokers.

References

1 Eurostat (2007) European statistic database. Available: <http://epp.eurostat.ec.europa.eu/portal/page/portal/health/public_health/database>. Accessed July 2010.

2 Mannino DM, Doherty DE, Sonia Buist A (2006) Global Initiative on Obstructive Lung Disease (GOLD) classification of lung disease and mortality: findings from the Atherosclerosis Risk in Communities (ARIC) study. Respir Med 100: 115-122.

3 Ekberg-Aronsson M, Pehrsson K, Nilsson JA, Nilsson PM, Lofdahl CG (2005) Mortality in GOLD stages of COPD and its dependence on symptoms of chronic bronchitis. Respir Res 6: 98.
